# Modeling of Ammunition Dynamic Pressure Measurement Chain in Ballistic Tests

**DOI:** 10.3390/s23198081

**Published:** 2023-09-26

**Authors:** Caio Bittencourt Cardoso Felix, Khrissy Aracélly Reis Medeiros, Carlos Roberto Hall Barbosa

**Affiliations:** 1 Postgraduate Programme in Metrology, Pontifical Catholic University of Rio de Janeiro, Marquês de São Vicente Street, 225, Gávea, Rio de Janeiro 22451-900, Brazil; caiobittencourt@gmail.com; 2 Mechanical Engineering Department, Optical Fiber Sensors Laboratory, Pontifical Catholic University of Rio de Janeiro, Marquês de São Vicente Street, 225, Gávea, Rio de Janeiro 22451-900, Brazil; kmedeiros@puc-rio.br

**Keywords:** equivalent circuit, impedance analysis, interior ballistics, pressure measurement, piezoelectric transducer

## Abstract

The use of piezoelectric transducers for internal dynamic pressure measurements in ammunition testing provides a significant advantage in the development and performance analysis of weapons and ammunition. Knowledge of the electrical characteristics of the dynamic pressure measurement chain, which includes the piezoelectric transducer and the charge amplifier, is a relevant condition for the design of interior ballistics pressure measurement systems. Thus, this study aims to characterize and model a piezoelectric transducer and its associated charge amplifier. First, the piezoelectric transducer was characterized using impedance analysis and modeled using a least squares curve-fitting tool, according to the Butterworth–Van Dyke model. Next, the charge amplifier was characterized through response analysis based on known inputs and modeled using LTSpice simulation techniques and the least squares curve-fit tool. Consequently, a measurement chain model is presented and simulated for two cases with different impulse signals. The first impulse signal was obtained from an interior ballistics computer simulation, and in the second case, it was considered the negative step signal characteristic of the calibration of piezoelectric transducers by means of dead weight. From the simulations, it was possible to verify the effectiveness of the model, which provided results with a low error in relation to the original pressure curve, and its applicability is demonstrated by the result of the simulation of the pressure variation in the calibration, where the attenuation of the signal can be visualized as the characteristic of the input curve changes.

## 1. Introduction

The piezoelectric effect was first discovered in 1880 by Pierre Curie and Jacques Curie [1]. However, its first application as a sensor was explored only in 1919 by J. J. Thomson, who reported that tourmaline or quartz crystals could be used in pressure measurements from explosions [2].

The direct piezoelectric effect is observed in specific materials, such as quartz crystals. This physical phenomenon can be described as the generation of an electrical charge in a material that has such properties when it is subjected to mechanical stress [3]. This effect has several applications, one of which is the use of piezoelectric transducers to measure dynamic pressure in different systems.

Thus, piezoelectric pressure transducers convert pressure into an electrical charge, allowing the measurement and monitoring of that physical quantity. These measuring instruments can be used in different contexts, such as the automotive, petrochemical, aerospace, ballistics and defense industries [4,5].

Notably, in ballistic testing, piezoelectric transducers are used to measure the internal pressure during the combustion process of an ammunition propellant, or even closed vessel tests focusing on determining the ballistic properties of the propellants. This measurement method is the most advantageous in several aspects [6]. Piezoelectric transducer measurements make it possible to obtain relevant information, such as the maximum pressure inside the chamber and the pressure profile over time, yielding helpful knowledge in the development of weapons and the analysis of the performance and safety of ammunition [7].

Measurements using piezoelectric transducers originate from the direct piezoelectric effect, an electrical charge on the order of picocoulombs (pCs), which must be converted to pressure by dividing it by the piezoelectric sensitivity coefficient, whose unit is pC/MPa. Because the charge generated is too small for direct measurement, to make pressure measurements possible using piezoelectric transducers, it is necessary to use charge amplifiers, which convert the electric charge originating from the direct piezoelectric effect into a voltage [8,9]. For example, in the case of the HPI B217 charge amplifier, the output voltage is from –10 V to +10 V, a voltage range with sufficient amplitude to be measured by any commercial oscilloscope.

Given this, the measurement of ammunition dynamic pressure in ballistics tests necessarily takes place by means of a measurement chain composed of a piezoelectric transducer and a charge amplifier. Therefore, modeling piezoelectric transducers and charge amplifiers as electrical circuits undoubtedly represents a valuable tool for simulations and theoretical studies of the dynamic pressure measurement chain, allowing a better understanding of how these devices work and how their electrical configuration affects dynamic pressure measurements in ammunition testing. As the signals obtained from dynamic pressure measurements are necessarily conditioned by charge amplifiers, the modeling of the two components of the measurement chain is relevant because it will ensure that the result of simulations provided by the model created is as reliable as possible for the real case. Additionally, no methodology with a similar objective, that is, modeling the measurement chain completely, was found in the literature.

Therefore, this study aimed to model the dynamic pressure measurement chain of interior ballistics. For this, two different methods were considered: (i) impedance analysis for the HPI GP6 piezoelectric transducer; and (ii) characterization for the HPI B217 charge amplifier, that is, the identification of the circuit parameters by means of the system response, given a known input. Furthermore, with the results, a simulation with ballistics data is implemented, aiming to demonstrate the applicability of the complete model.

This paper is divided as follows: Section 2 briefly discusses the theoretical foundation of the metrological core of the study. Section 3 presents the methods used for the modeling. Section 4 shows the results obtained, that is, the elements of each electrical circuit model of the piezoelectric transducer and the charge amplifier, as well as examples of the application of the model with the pressure curve of interior ballistics obtained by simulation and with pressure curves characteristic of the calibration of piezoelectric transducers using dead weight. Finally, Section 5 presents the conclusions and final considerations of the study.

## 2. Interior Ballistics—Internal Pressure Measurement

Piezoelectric transducers play a crucial role in ensuring metrological reliability during weapons and ammunition testing, because the most critical parameter to measure is the internal pressure resulting from the expansion of the propellant’s combustion gases [10]. These transducers allow for the measurement of the pressure profile developed by the ammunition, enabling the accurate evaluation of weapon and ammunition performance and safety. Therefore, piezoelectric transducers are essential devices for testing weapons and ammunition.

In ammunition tests in which the internal pressure is measured, one or two piezoelectric transducers can be installed, with the positions defined by each certifying organization. For EPVAT tests (Electronic Pressure Velocity and Action Time), the North Atlantic Treaty Organization (NATO) determines that a transducer is installed in the test barrel in a position equivalent to the mouth of the ammunition case for tests of all calibers, aiming to measure the “case mouth pressure”, and in the case of calibers of 5.56 × 45 mm and 7.62 × 51 mm, a transducer should also be installed close to the intermediate region of the test barrel for the measurement of “port pressure” [11].

**Figure 1:**
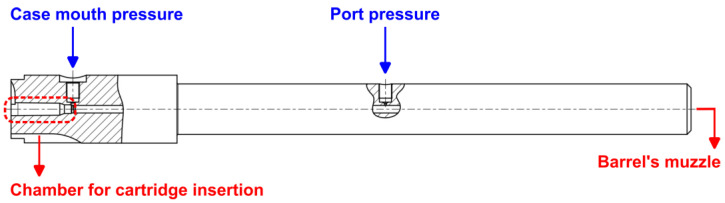
Test barrel with two transducer mounting ports for EPVAT test.

Figure 1 shows the test barrel with two transducer mounting ports. For the EPVAT tests, the HPI model GP6 transducer (HPI GP6) can be used for both case mouth and port pressure measurements.

Piezoelectric sensors are classified as active, that is, they do not require an external power source to perform measurements [12]. Piezoelectric transducers used in ballistics tests generate an electrical charge as their sensitive material, a piezoelectric crystal, is compressed by the effect of increased pressure inside the barrel.

**Figure 2:**
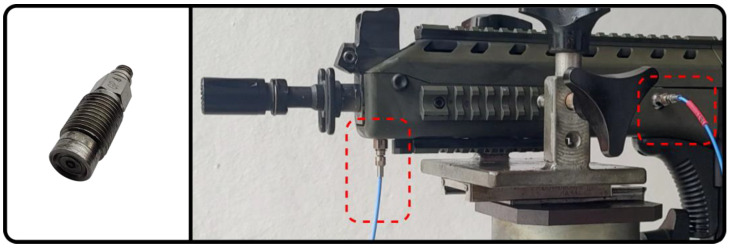
HPI GP6 pressure transducer and an example of use to measure internal barrel pressure of Imbel IA2 5.56 × 45 mm.

The HPI GP6 transducer, shown in Figure 2, used in this study, has a gallium phosphate (GaPO4) sensitive element, a synthetic piezoelectric crystal derived from quartz. This material has a higher sensitivity than quartz, which is the sensitive element of other piezoelectric pressure transducers used in ballistics tests [13]. As quartz, the gallium phosphate used in pressure transducers works ideally for dynamic measurements, i.e., it generates an electrical charge as pressure is applied to or removed from it. If static pressure is applied, the charge quickly leaks away, and the transducer response returns to zero [14].

The HPI GP6 transducer measures dynamic pressures of up to 600 MPa, has a natural vibration frequency greater than 240 kHz and, in the case of the unit used in the tests described in this document, has a nominal sensitivity of 33 pC/MPa.

**Figure 3:**
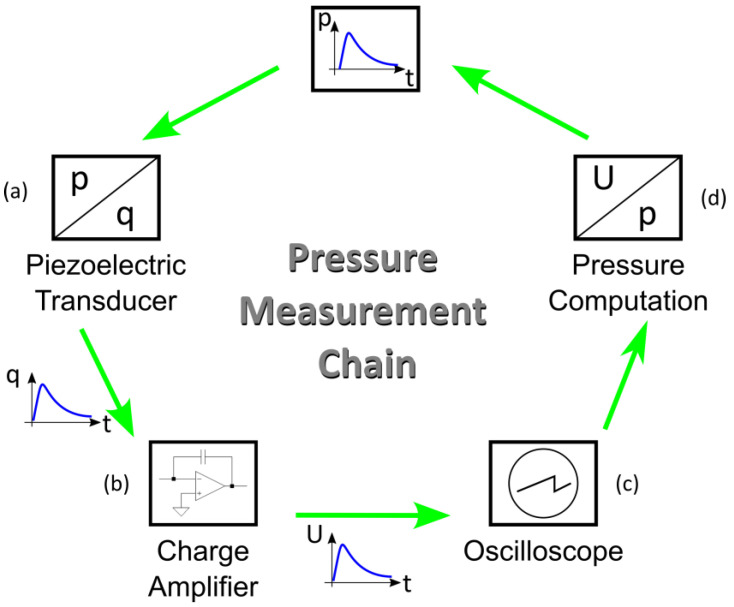
Measuring chain for pressure using a piezoelectric transducer: (**a**) pressure is converted to electric charge by the piezoelectric transducer; (**b**) electric charge is converted to voltage by the charge amplifier; (**c**) voltage is read by an oscilloscope; (**d**) knowing the sensibility of the piezoelectric transducer and the gain of the charge amplifier, pressure is computed. Adapted from [15].

To obtain accurate and reliable readings from piezoelectric transducers, it is necessary to use charge amplifiers in the instrumentation to convert the electric charge originating from the direct piezoelectric effect into a proportional electric voltage. Thus, from the dynamic pressure pulse, the piezoelectric transducer produces an electric charge that, in turn, is converted into a voltage by the charge amplifier. Finally, the voltage measurement can be performed using an oscilloscope, for example, so that it is converted into pressure, knowing the charge amplifier amplification factor and the sensitivity of the piezoelectric transducer, completing the pressure measurement chain, summarized in Figure 3.

In this study, the charge amplifier used was the HPI B217, which provides voltage as an output parameter, or even pressure over time, employing its control and parameter reading software, the HPI B3000. The pressure curve over time is determined using the HPI B217 charge amplifier’s amplification factor, which will provide the charge measurement, and, finally, with the piezoelectric sensitivity of the HPI GP6 transducer, convert the charge to pressure.

**Figure 4:**
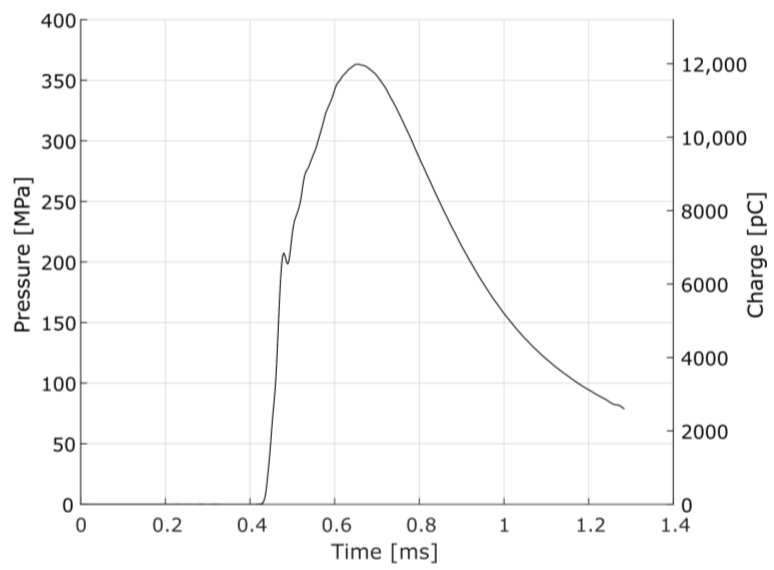
Case mouth pressure-time variation in EPVAT test barrel for 7.62 × 51 mm NATO Ball ammunition: pressure can be obtained by dividing the measured charge by the transducer sensitivity (33 pC/MPa), considering a linear behavior.

The measurement chain provides the reading of the pressure pulse inside the barrel, which has the particularity of high-pressure levels reached in a few milliseconds [16,17]. A typical pressure-time curve that characterizes the interior ballistics of ammunition is shown in Figure 4. In that curve, which is the measurement of the pressure variation between the ignition of the primer and the output of the projectile by the barrel, the maximum pressure of approximately 363 MPa is verified, reached at 0.240 ms after the beginning of the pressure variation, in the case of a pressure test of 7.62 × 51 mm NATO Ball ammunition, measured at the case mouth port and filtered by a 10 kHz 2nd-order Butterworth low-pass filter, as recommended by the experimental procedure.

The modeling of the entire measuring chain in equivalent electronic circuits, that is, the modeling of piezoelectric transducers and charge amplifiers, provides a better understanding of the electrical characteristics of piezoelectric transducers. Therefore, it is essential for the design of measurement systems based on piezoelectric sensors [18].

## 3. Modeling of the Interior Ballistics Pressure Measurement Chain

To obtain the electrical model of the entire measurement chain, the methods used in modeling the piezoelectric transducer, by impedance analysis, and then for the charge amplifier, using the characterization method, will be presented.

### 3.1. Piezoelectric Transducer Modeling Subsection

**Figure 5:**
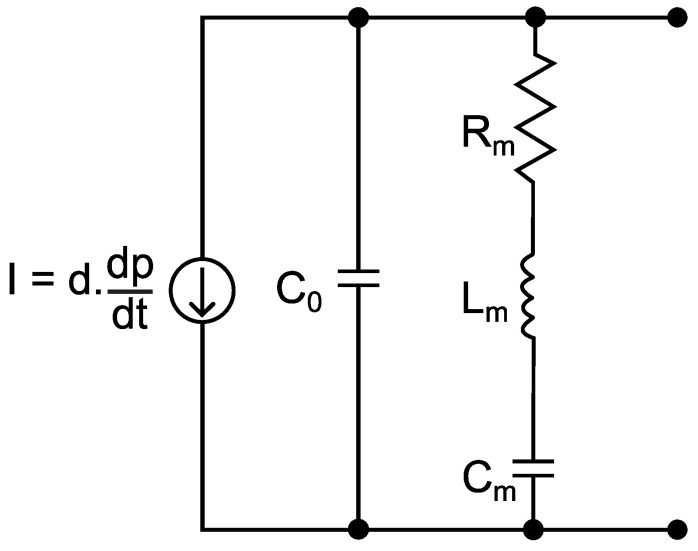
Butterworth–Van Dyke electric model.

For the modeling of the HPI GP6 piezoelectric transducer, the model called Butterworth–Van Dyke (BVD) was considered, as recommended by the IEEE Standard on Piezoelectricity (ANSI/IEEE Std 176-1987) [19]. The BVD model is useful for the analysis of the electrical performance of piezoelectric transducers [20] and can be used in the design and optimization of piezoelectric devices [21,22,23,24]. The BVD model circuit consists of a resistor, an inductor and a capacitor in series ($R_{m}$, $L_{m}$, $C_{m}$), all connected in parallel with a second capacitor ($C_{0}$), as shown in Figure 5.

The capacitance $C_{0}$, which dominates the transducer impedance outside the resonance region, models the piezoelectric elements’ dielectric capacitance and the parasitic capacitance of the cables, whereas the parameters $R_{m}$, $L_{m}$ and $C_{m}$ model the mechanical oscillation of the transducer [25].

For piezoelectric transducers used in ballistics tests, the magnitude to be measured is the dynamic pressure developed inside the barrel of a weapon. Therefore, pressure must be the input data of the circuit. In turn, the electrical charge (q) is defined by the piezoelectric sensitivity (d) of the transducer, which defines the relationship between the electrical charge and the pressure. Thus, in the electrical model, the input data will be represented by a current source (I), which will be determined by the derivative of the electrical charge with respect to time (dQ/dt), or by the derivative of the pressure in time (dP/dt), multiplied by the piezoelectric sensitivity (d):

(1) I=dQdt=d·dPdt

To determine the BVD model’s parameters, the following technique will be used: the impedance of the BVD model ($Z_{BVD}$) will be fitted to the characteristic impedance of the piezoelectric transducer, measured experimentally. For the BVD model, the $Z_{BVD}$ impedance is calculated by:

(2) ZBVD=(1jωC0)(jωLm+Rm+1jωCm)1jωC0+jωLm+Rm+1jωCm

**Figure 6:**
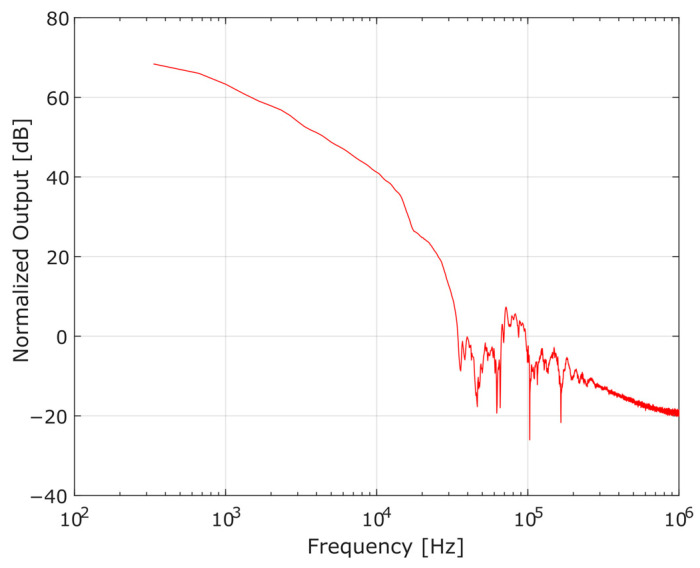
Fast Fourier Transform of the pressure-time signal shown in Figure 4.

From the impedance equation of the BVD model (2), its elements can be determined employing the least squares curve fitting of the impedance obtained experimentally, unlike other approaches, in which the determination of the elements of the electric circuit takes place by means of parametric equations dependent on the magnitude of the impedance at specific frequencies [22,24,25], or even by employing physical parameters that are characteristic of the piezoelectric material of the transducer [26]. For this, we used a Keysight E4900A impedance analyzer, which basically excites the transducer through a voltage of 100 mV with a variable frequency and measures its response, providing the reactance and resistance for a frequency range from 1 kHz to 100 kHz. Calculated by Fast Fourier Transform (FFT), the frequency domain representation of the pressure-time signal of Figure 4 is shown in Figure 6, showing that the useful signal is lower than 20 kHz. So, it is acceptable to use the referred frequency range (1–100 kHz) for the impedance fit.

**Figure 7:**
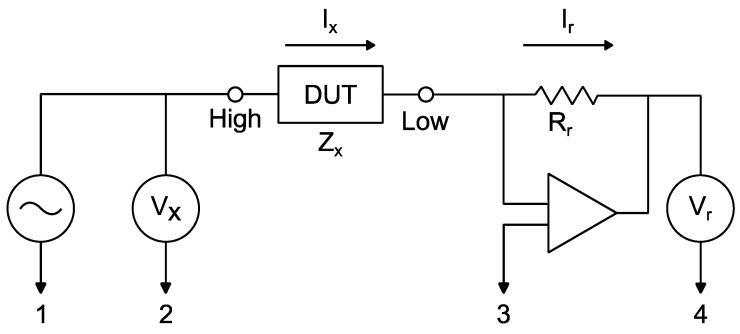
Impedance measurement circuit: Auto-Balancing Bridge Method [27].

For impedance measurements by an impedance analyzer, some connection circuits can be used, depending on many factors, such as the frequency range to be examined. In this case, the circuit detailed in Figure 7, called the Auto-Balancing Bridge Method, was used.

In the measuring circuit, the current $I_{x}$ passes through the device under test (DUT). In turn, the current $I_{r}$, which passes through resistor $R_{r}$, is converted into voltage $V_{x}$, measured by terminal 4.

(3) VxZx=Ix=Ir=VrRr

Since the current $I_{r}$ is equal to $I_{x}$, the impedance $Z_{x}$ is determined by the $V_{x}$ and $V_{r}$ voltages, measured by terminals 2 and 4, respectively [27], that is:

(4) Zx=Rr·VxVr

Based on the results of the impedance analysis, the function “lsqcurvefit”, available in MATLAB (version 9.13.0.2105380), was used to determine the parameters of the BVD model for the piezoelectric transducer HPI GP6. The function receives as input parameters the nonlinear function determined by (2), as well as the experimental data to which it must be fitted, in addition to the initial values and the upper and lower limits for the variables to be estimated.

### 3.2. Charge Amplifier Modeling

**Figure 8:**
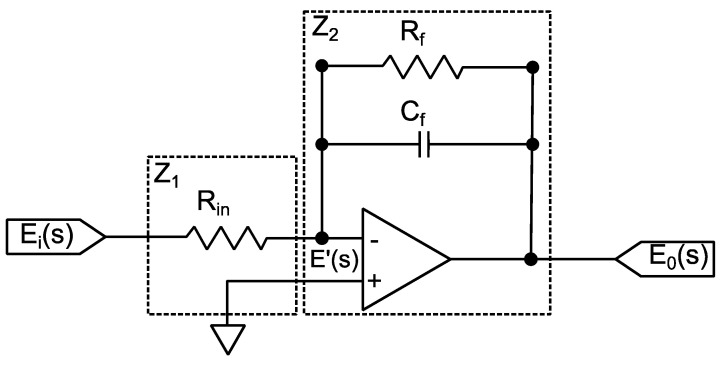
Charge amplifier electrical model.

Advancing in the modeling of the dynamic pressure measurement chain, in the case of the charge amplifier, the simplified topology presented in Figure 8 [28,29,30,31] was adopted to compose the modeling as a whole.

In this circuit model, the transfer function is derived by:

(5) $$\frac{E_{0}\left(s\right)}{E_{i}\left(s\right)}=-\frac{Z_{2}\left(s\right)}{Z_{1}\left(s\right)\;}=-\frac{1/R_{in}C_{f}}{s+1/C_{f}R_{f}}$$

Applying the inverse Laplace transform to the transfer function, the following relationship between the output voltage and the input voltage is obtained:

(6) $$\frac{e_{o}\left(t\right)}{e_{i}\left(t\right)}=-\frac{1}{R_{in}C_{f}}\cdot e^{-\frac{1}{R_{f}C_{f}}t}$$

Upon the input of a step signal, the initial output voltage will be $-1/R_{in}C_{f}$, returning to zero according to the exponential function with the time constant $\tau =C_{f}R_{f}$. The limit for the duration of charge measurements can be defined in the interval 0<t<0.02τ, for an error limit of 2%, for example [12].

**Figure 9:**
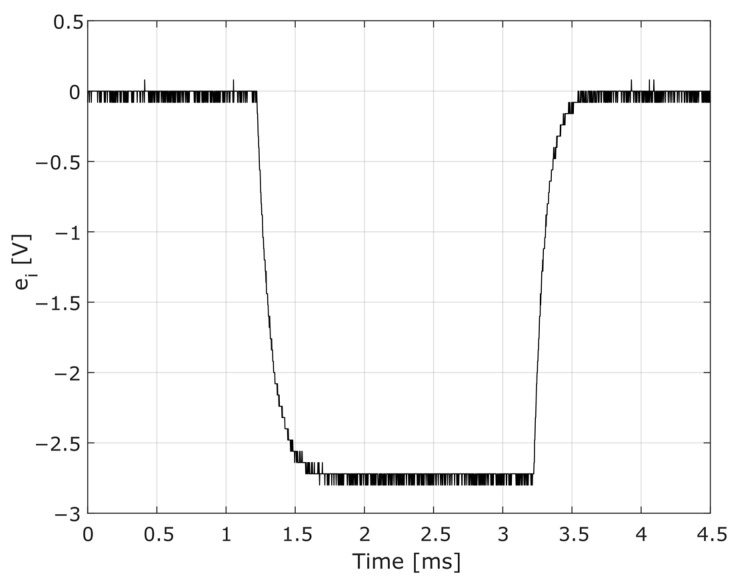
Input curve generated from HPI B202 for characterization of the charge amplifier.

Intending to characterize the HPI B217 charge amplifier, including allowing the determination of the time constant, the following procedure was adopted: from a previously defined input voltage, the amplifier response was observed using the digital oscilloscope Tektronix model TBS 1102B. The input was established using the HPI B202 function generator, as shown in Figure 9. The instrument generated a negative rectangular curve, $e_{i}\left(t\right),$ with a minimum amplitude of approximately −2.7 V.

Plugged into the output of the function generator, a reference capacitor with capacitance $C_{ref}$ was inserted, allowing the voltage $e_{i}\left(t\right)$ to be converted into the electric charge q(t), according to:

(7) $$q\left(t\right)=C_{ref}\cdot e_{i}\left(t\right)$$

Subsequently, the amplifier was connected in series to the circuit, and its response was observed with the same oscilloscope mentioned above. For the characterization, four different reference capacitors were used, with the respective capacitances described in Table 1.

**Figure 10:**
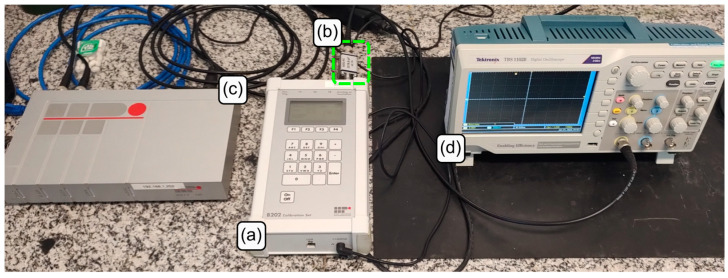
Instruments series connection: (**a**) function generator HPI B202; (**b**) reference capacitor; (**c**) charge amplifier HPI B217; and (**d**) oscilloscope.

Figure 10 shows the instruments connected in series for the characterization of the HPI B217 charge amplifier.

**Figure 11:**
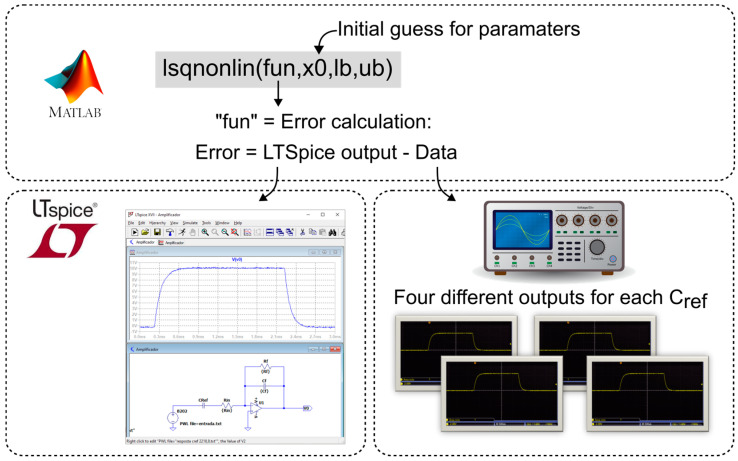
Charge amplifier characterization routine.

From the different responses obtained in the characterization, similar to the piezoelectric transducer modeling, a MATLAB curve-fitting tool was used. This time, the function used was “lsqnonlin”, which also fits the parameters vector to a nonlinear function. In this case, the MATLAB function fits the data measured by the oscilloscope to the data simulated by a circuit model implemented in LTSpice (version 17.0.36.0), that is, the corresponding parameters (the values of the components in Figure 8) were inserted into LTspice, and the simulated result was compared to the input data obtained with the oscilloscope, thus determining the error. Figure 11 illustrates the routine developed for the charge amplifier characterization.

### 3.3. Parameters Refinement

For now, the data fitting made took into account the behavior of the measurement chain components separately, that is, the data fitting of the electrical model of the piezoelectric transducer (Figure 5) to the data originated with the impedance analysis, as well as the data fitting of the respective model (Figure 8) to the charge amplifier characterization data.

Since this study deals with a measurement chain modeling composed of the HPI GP6 piezoelectric transducer and the HPI B217 charge amplifier, it is possible to perform the data fitting considering the complete electrical model. For this, it is necessary to determine the input and the expected response data of the circuit. Considering the experimental pressure/electric charge curve, illustrated in Figure 4, and assuming that it is identical to the input of the piezoelectric transducer, that is, that it faithfully represents the phenomenon of internal ballistics characterized in the pressure curve over time, it is desirable that the measurement chain response is identical to the input. Therefore, aiming at a refinement of the parameters, with the objective of reducing the response error of the electrical model developed, a data fitting of the electrical model of the measurement chain to the experimental data illustrated in Figure 4 was carried out.

**Figure 12:**
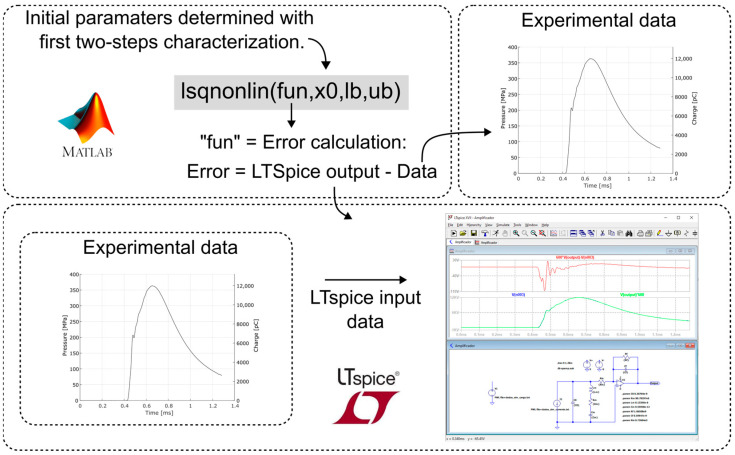
Parameters refinement routine.

Similar to the characterization of the charge amplifier, using MATLAB’s “lsqnonlin” function, the parameters were fitted to the data simulated by the electrical model implemented in LTspice, that is, the parameters of the complete model of the measurement chain were inserted in LTspice, and the simulation results were compared with the experimental data, identical to the model input data, thus determining the error. In this case, the initial values of the seven parameters were those obtained in the first two steps of the modeling, that is, in the characterization of the piezoelectric transducer and then in the characterization of the charge amplifier. Figure 12 illustrates the refinement routine of the parameters determined in the characterizations.

**Figure 13:**
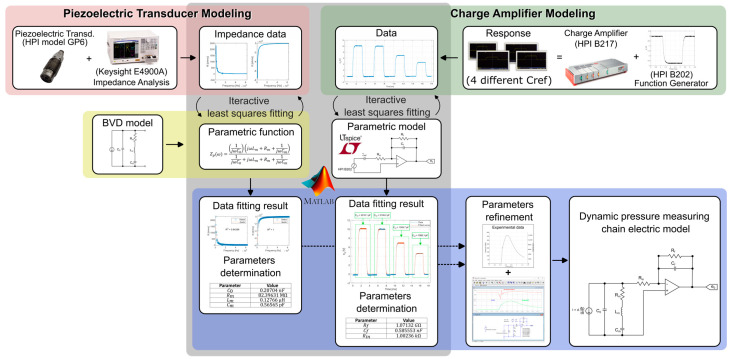
Modeling of dynamic pressure measuring chain.

Finally, the three-step modeling is completed, starting with the piezoelectric transducer and the charge amplifier modeling, and ending with parameters refinement. Figure 13 illustrates the entire modeling process, culminating in the electrical model of the dynamic pressure measurement chain.

### 3.4. Simulations Applied to Pressure Measurement in Calibration

To demonstrate the applicability of the complete model, two sequential simulations were performed. The first uses data obtained with the interior ballistics module of PRODAS software (Projectile Rocket Ordnance Design and Analysis System), which simulates the pressure gradient inside the barrel based on empirical functions [32]. Basically, PRODAS provides the temporal evolution of displacement, pressure and velocity for a projectile inside the barrel, based on characteristics of the primer and propellant, projectile and weapon, that is, dimensions of the chamber, rifling and barrel length, in addition to maximum pressure data inside the barrel and the final velocity of the projectile (muzzle velocity) [33].

Then, a second simulation was performed using data from a hypothetical calibration. The calibration of piezoelectric transducers similar to HPI GP6, according to the standards for the evaluation of ammunition [11,34], can be performed using a dead weight, a system intended for the direct calibration of static pressure measuring instruments. Since piezoelectric transducers are not able to measure static pressures, a valve system with rapid pressure relief is coupled to the dead weight, inserting into the calibration process a dynamic event that can be characterized as a negative step [16]. Therefore, in the case of the calibration simulation, the negative step was modeled according to the sigmoid function [32], whose expression is determined by:

(8) $$q\left(t\right)=d\cdot P_{max}\cdot \left(1-\frac{1}{1+e^{-2kt}}\right)$$

where d is the piezoelectric sensitivity; $P_{max}$ is the maximum pressure obtained in the calibration, determined by the dead weight; and k is the coefficient that will determine the duration of the dynamic event.

For the calibration of piezoelectric transducers, the duration of the dynamic event is directly linked to the characteristics of the charge amplifier, as already mentioned in Section 3.2. Thus, there is relevance in simulating step inputs with varying fall times (defined between the levels of 90% and 10% of the maximum charge) in order to determine the limit for the correct measurement of the pressure/electric charge, without attenuation of the response caused by the measurement chain. Thus, by varying the constant k, it is possible to create different dynamic events with their respective fall times, as shown in Table 2.

In both simulations, the current (I), input to the electrical model of the pressure measurement chain, was calculated by the discrete derivative of pressure (P) with respect to time, which in turn was calculated by the piezoelectric sensitivity (d) of 33 pC/MPa, according to Equation (1).

## 4. Results

### 4.1. HPI GP6 Piezoelectric Transducer Electric Model

**Figure 14:**
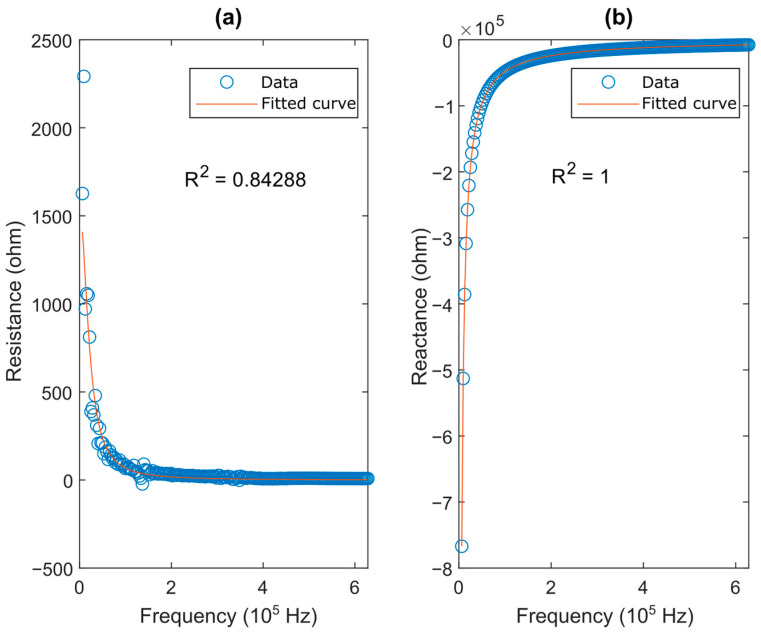
Result of data fitting for BVD model: (**a**) resistance and (**b**) reactance.

After measuring the impedance of the HPI GP6 piezoelectric transducer with the impedance analyzer, and considering the BVD model impedance equation presented in Figure 5, by using the methodology detailed in Section 3.1, the curve fitting in MATLAB using the “lsqcurvefit” function could be obtained as shown in Figure 14, with the optimized parameters shown in Table 3.

To verify the feasibility of the BVD model in simulations, the equivalent electrical circuit model for the charge amplifier will first be determined, and then it will be observed the response of the measurement chain electrical model.

### 4.2. HPI B217 Charge Amplifier Electric Model

**Figure 15:**
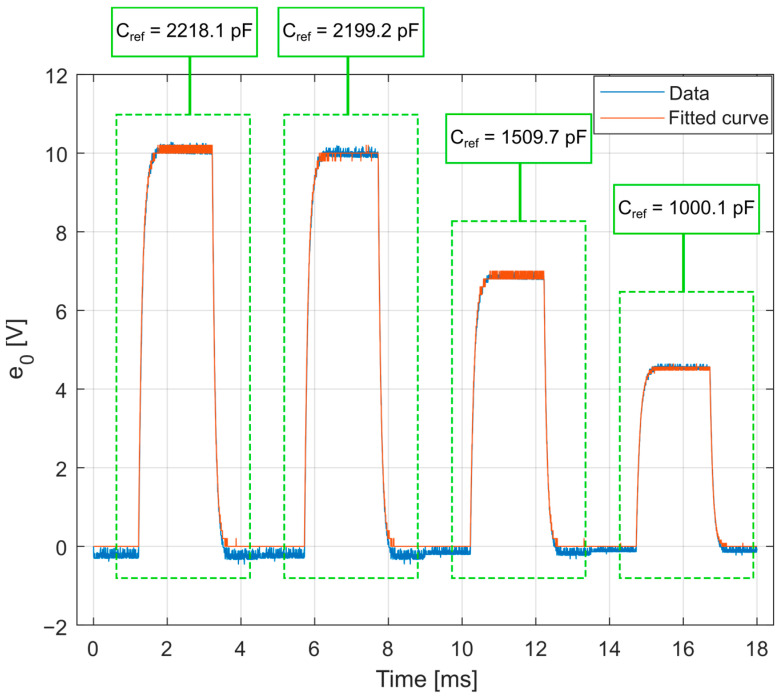
Results of the series of data fitting for each reference capacitor, which compose the equivalent circuit modeling of the HPI B217 charge amplifier.

By means of the procedure described in Section 3.2, using the curve-fitting procedure to obtain the charge amplifier circuit response from the known input, it is possible to determine the desired parameters of the HPI B217 charge amplifier electric model. Figure 15 shows the curve-fitting result, considering the four outputs for each reference capacitor.

The obtained parameters are presented in Table 4. For the curve fit, the determination coefficient was 0.9984, and the charge amplifier time constant was calculated as τ=0.6273 s. The amplification factor of the HPI B217 charge amplifier was 1.667 mV/pC, defined by the relationship between the maximum response voltage and the input charge.

### 4.3. Parameters Refinement

**Figure 16:**
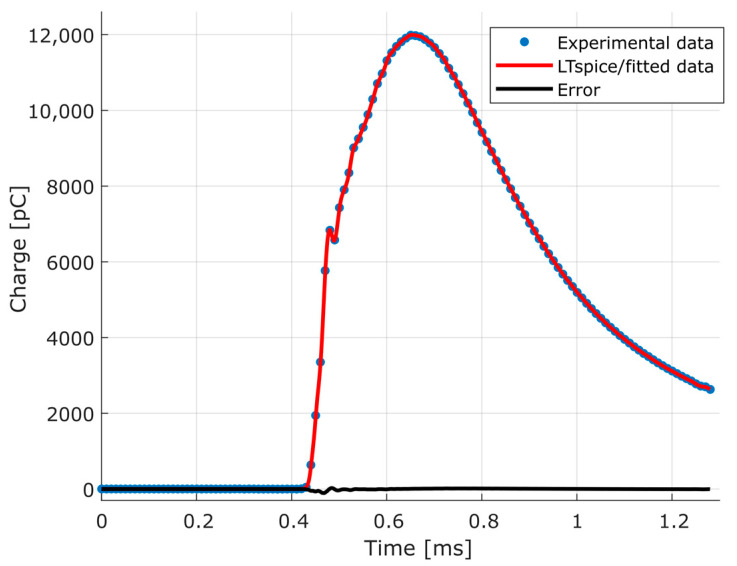
Result of the refinement of the parameters determined by means of the experimental data, and the calculated error.

After modeling each element of the dynamic pressure measurement chain, the refinement of the parameters described in Section 3.3 was carried out. Using the experimental data illustrated in Figure 4, the electrical model was fitted, obtaining the response through simulation in LTspice, as shown in Figure 16. For the obtained data fitting, the determination coefficient was 0.9999.

**Figure 17:**
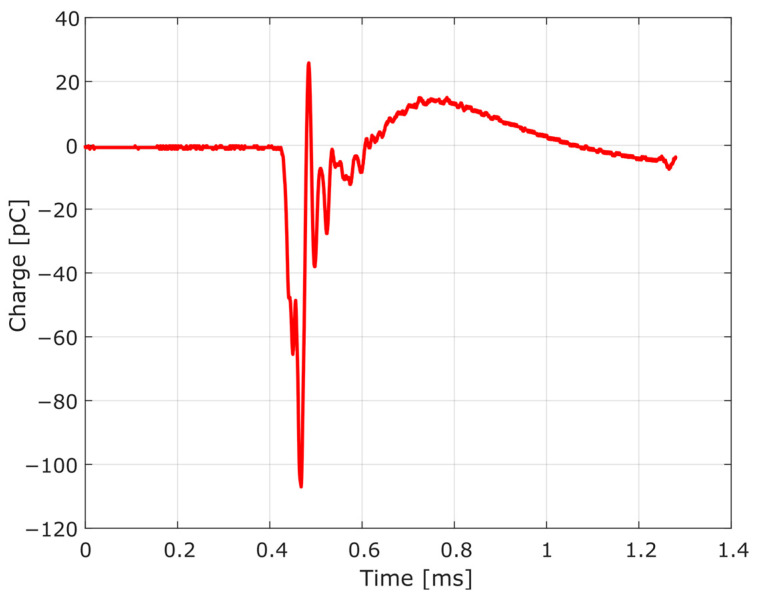
Error calculated between experimental data and simulated data obtained with refined parameters.

The calculated error can be best seen in Figure 17, with the error at the point where the maximum electric charge is obtained as 6.89511 pC (0.0575%).

**Figure 18:**
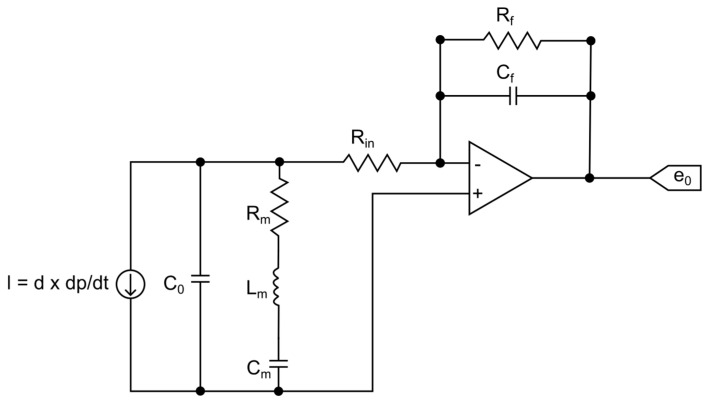
Dynamic pressure measuring chain electrical model composed by piezoelectric transducer and charge amplifier.

Thus, with the modeling of the HPI GP6 piezoelectric transducer and the HPI B217 charge amplifier, after the parameters refinement with experimental data, it is possible to compose the entire pressure measurement chain and present the complete circuit, as shown in Figure 18.

The values of each parameter obtained in the two least squares curve-fit procedures, followed by the refinement of the entire parameter set, are shown in Table 5.

Regarding the modeling of the measurement chain, it is important to highlight that it was based on the impedance analysis and the response of the charge amplifier, aiming at electrical modeling of the electromechanical components (namely, the piezoelectric transducer and charge amplifier), and not of the physics behind the generation of the pressure curve originating with the interior ballistics. Therefore, aspects relating to interior ballistics, such as design values of weapons and ammunition and the energy characteristics of the gun propellant, are not considered. In other words, the generation of the pressure curve P(t) was not the objective of the modeling, but the curve itself was used as input to improve the electrical model of the measurement chain.

### 4.4. Simulation of Interior Ballistics Pressure Measurement

After determining the measurement chain equivalent circuit, that is, the modeling of the HPI GP6 piezoelectric transducer and the HPI B217 charge amplifier, in order to demonstrate the applicability of the complete model, simulations were performed with interior ballistics data in LTspice, according to the methodology described in Section 3.

**Figure 19:**
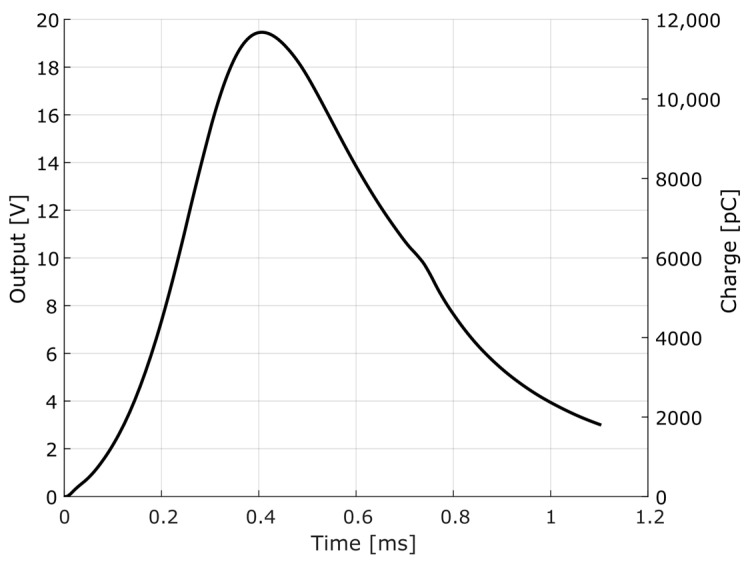
Theoretical breech pressure-time curve for 7.62 × 51 mm NATO Ball ammunition in EPVAT test barrel.

In order to generate input data for the first simulation, that is, the pressure-time curve, the PRODAS interior ballistics module was used. In this example, the parameters of the 7.62 × 51 mm NATO Ball ammunition were adopted, with data from a 7.62 × 51 mm EPVAT test barrel. Figure 19 shows the theoretical breech pressure curve over time for the 7.62 × 51 mm NATO Ball caliber simulation, as well as the calculated electrical charge for a transducer with a nominal sensitivity of 33 pC/MPa.

As the current is the derivative of the electric charge with respect to time, it is possible to determine it from the charge-time curve. This, therefore, will be the input of the pressure measurement chain electric model, to be simulated by LTspice.

**Figure 20:**
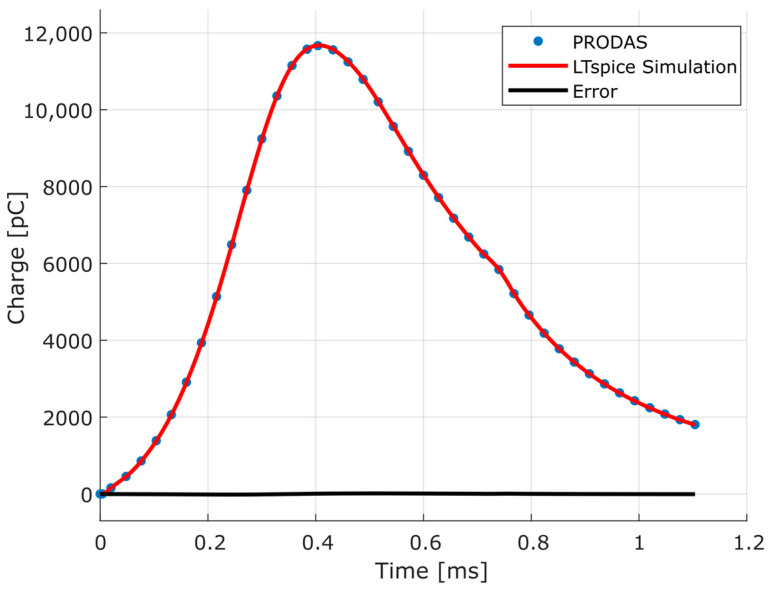
Charge-time curve of PRODAS simulation and LTspice circuit model simulation, and calculated error between them.

From the assembly of the electrical circuit in LTspice and the data from simulation of interior ballistics obtained with PRODAS, it was possible to obtain the results shown in Figure 20.

**Figure 21:**
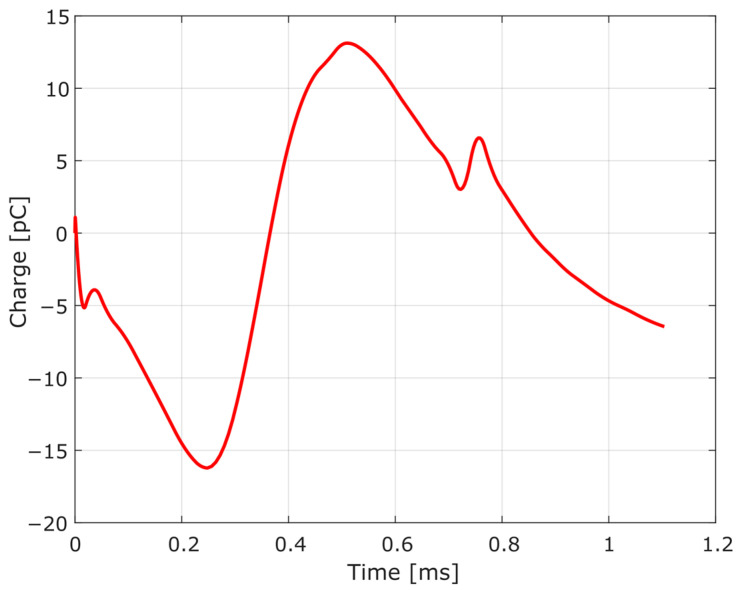
Calculated error between charge-time curve of PRODAS simulation and LTspice circuit model simulation.

The calculated error can be better seen in Figure 21. The error corresponding to the maximum pressure point is 7.6140 pC (0.0653%).

For the simulation of the calibration by means of dead weight together with the pressure relief system, seven characteristic curves of the negative step of the pressure/electric charge were used, based on the expression (8), by varying the constant k according to Table 2.

**Figure 22:**
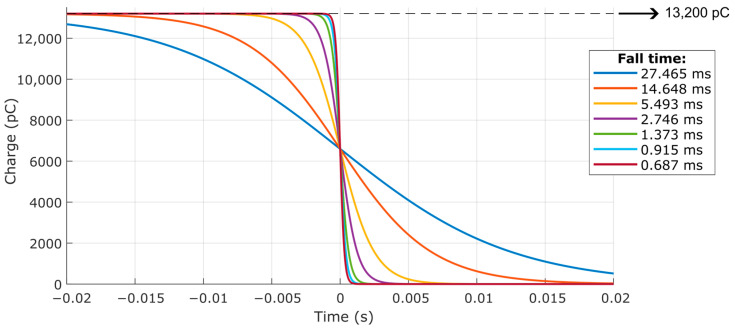
Input signal with different durations for calibration simulation.

For a calibration pressure $(P_{max}$) of 400 MPa, assuming the piezoelectric sensitivity (d) of 33 pC/MPa, the different inputs can be determined according to Figure 22. Each input signal is a negative step with an initial electric charge of 13,200 pC, corresponding to 400 MPa, with the different fall times of the dynamic event, with a final electric charge of 0 pC, corresponding to 0 MPa.

**Figure 23:**
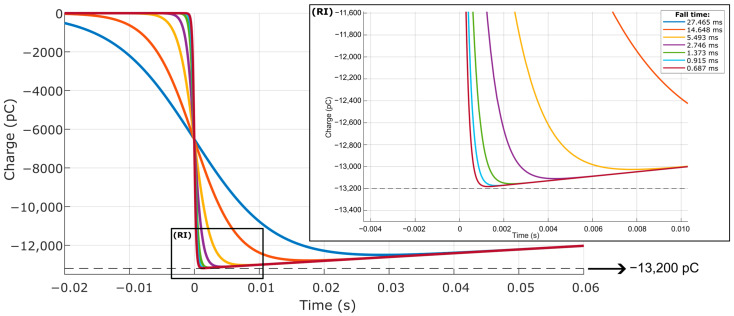
Output signal with different durations originated with calibration simulation, focused on minimum charge values (RI).

After differentiating the negative step signals with respect to time, an operation necessary for the use of the different input signals in the current source of the electrical model, the seven calibration simulations were performed, resulting in the responses illustrated in Figure 23, which highlights the Region of Interest (RI), in which the different responses reach their respective minimum values of electric charge. Since the pressure step produces a negative pressure variation, the response signal has negative values. Therefore, the maximum expected charge is −13,200 pC.

Analyzing the responses, it is noticed that the slower the dynamic event, that is, the opening of the valve system represented by the negative step inserted in the model, the greater the attenuation of the input signal by the measurement chain. In all cases, after reaching the respective minimum values, the electric charge curves follow the characteristic discharge curve of the modeled charge amplifier. Table 6 details, for each electric charge curve originated with the simulation, the difference between the desired load, i.e., −13,200 pC, and the minimum load obtained, in addition to the equivalent pressure, considering the piezoelectric sensitivity of 33 pC/MPa.

## 5. Conclusions

Fundamentally, this work starts from the assumption that a better understanding of the electrical characteristics of piezoelectric transducers can be obtained from the modeling of the entire measuring chain in equivalent electronic circuits. Thus, the interior ballistics pressure measurement chain of ammunition was modeled by an electronic circuit.

For this purpose, the HPI GP6 piezoelectric transducer and the HPI B217 charge amplifier, both used to measure dynamic pressure in ballistics tests of ammunition, were modeled. As an initial step for the HPI GP6 modeling, the BVD model for the piezoelectric transducer was determined from its characteristic impedance, obtained with an impedance analyzer, and the curve fit for the equivalent circuit was solved using MATLAB.

With regard to the charge amplifier, the characterization occurred based on the observation of its response to input variation, with the curve fit performed again with the help of MATLAB, this time in conjunction with LTspice software. Finally, the parameters were refined using the experimental pressure-time curve.

In addition, from the determination of the measurement chain circuit, that is, the transducer in series with the charge amplifier, two simulations were performed. First, the data obtained with the PRODAS interior ballistics module were used, resulting in an error at the maximum pressure point of 7.6140 pC (0.0653%).

In view of the results obtained, both in the refinement of the parameters through the experimental data and in the simulation with the data from PRODAS, the dynamic pressure measurement chain modeling was considered effective since the operations of multiplication by piezoelectric sensitivity and the derivation to obtain the current, followed by the simulation of the piezoelectric transducer and the charge amplifier electric models and, finally, the conversion of the output voltage in electric charge/pressure, return to the original curve without significant distortions.

Next, the calibration of the piezoelectric transducer was simulated using a dead weight with pressure variations with different fall times, represented by negative steps, as presented in Figure 22. In this case, the simulations demonstrated the attenuation of the input signals by the modeled measurement chain, indicating that the slower the dynamic event of pressure variation, the greater the difference between the input pressure and the measurement chain response.

In short, it was concluded that the calibration simulation of the piezoelectric transducer demonstrates the applicability of the generated model, and the information related to the attenuation of the signal by the measurement chain is relevant for studies aimed at the construction of calibration systems.

Regarding the applicability of the model to other piezoelectric transducers used in ballistics tests, it is believed that the methodology is applicable, given the similarity of the measurement chain. However, this statement requires scientific confirmation, which will provide comparability between the electrical models of different piezoelectric transducers.

Finally, it was also observed that the effectiveness of the modeling can be verified together with other applications aimed at testing propellants and ammunition, such as the piezoelectric transducer calibration method, called ‘dropweight’, and closed vessel tests. In this sense, this observation is an interesting suggestion for future work.

## Figures and Tables

**Table 1:** Reference Capacitors Values.

$C_{ref1}$	$C_{ref2}$	$C_{ref3}$	$C_{ref4}$
2218.8 pF	2199.2 pF	1509.7 pF	1000.1 pF

**Table 2:** Dynamic events fall times for each value of *k*.

*k* = 80	*k* = 150	*k* = 400	*k* = 800	*k* = 1600	*k* = 2400	*k* = 3200
27.465 ms	14.648 ms	5.493 ms	2.746 ms	1.373 ms	0.915 ms	0.687 ms

**Table 3:** BVD model parameters determined for the HPI GP6 transducer.

Parameter	Value
$C_{0}$	0.20704 nF
$R_{m}$	82.39631 MΩ
$L_{m}$	0.12766 μH
$C_{m}$	0.56565 pF

**Table 4:** Determined parameters for the HPI B217 charge amplifier.

Parameter	Value
$R_{f}$	1.07132 GΩ
$C_{f}$	0.58555 nF
$R_{in}$	1.00236 kΩ

**Table 5:** Parameters of the electrical model of the dynamic pressure measurement chain.

Parameter	Value
$C_{0}$	0.20704 nF
$R_{m}$	80.78297 MΩ
$L_{m}$	0.13356 μH
$C_{m}$	0.55958 pF
$R_{f}$	1.06508 GΩ
$C_{f}$	0.59947 nF
$R_{in}$	0.72604 kΩ

**Table 6:** Error for each signal with respective fall time (duration of dynamic event).

*k*	Fall Time	Error
*k* = 80	27.465 ms	700.6 pC/21.23 MPa (5.31%)
*k* = 150	14.648 ms	414.2 pC/12.55 MPa (3.14%)
*k* = 400	5.493 ms	174.5 pC/5.29 MPa (1.32%)
*k* = 800	2.746 ms	90.7 pC/2.75 MPa (0.69%)
*k* = 1600	1.373 ms	44.1 pC/1.34 MPa (0.33%)
*k* = 2400	0.915 ms	27.3 pC/0.83 MPa (0.21%)
*k* = 3200	0.687 ms	18.5 pC/0.56 MPa (0.14%)

## Data Availability

The data presented in this study are available upon request from the corresponding author. The data are not publicly available due to this study being part of a master’s degree research which is still in the completion phase.
